# Social Dominance Orientation, Dispositional Empathy, and Need for Cognitive Closure Moderate the Impact of Empathy-Skills Training, but Not Patient Contact, on Medical Students' Negative Attitudes toward Higher-Weight Patients

**DOI:** 10.3389/fpsyg.2017.00504

**Published:** 2017-04-04

**Authors:** Angela Meadows, Suzanne Higgs, Sara E. Burke, John F. Dovidio, Michelle van Ryn, Sean M. Phelan

**Affiliations:** ^1^School of Psychology, University of BirminghamBirmingham, UK; ^2^Department of Psychology, Yale UniversityNew Haven, CT, USA; ^3^Division of Health Care Policy and Research, Mayo ClinicRochester, MN, USA

**Keywords:** weight stigma, anti-fat attitudes, medical education, physician-patient relations, empathy, perspective taking, contact, individual differences

## Abstract

Anti-fat bias in healthcare providers and medical students has serious implications for quality of care of higher-weight patients. Studies of interventions aimed at reducing anti-fat attitudes in medical students have generally been disappointing, with little enduring effect. It is possible that some students may be more receptive to prejudice-reducing influences than others, due to underlying differences in their personal characteristics. It is also possible that attitudes toward patients, specifically, may differ from anti-fat attitudes in general, and prejudice-reduction effectiveness on patient-specific attitudes has not yet been evaluated. The present study explored the effect on general and patient-specific anti-fat attitudes of (1) contact with higher-weight individuals prior to and during medical school; and (2) training designed to increase medical students' empathy toward patients by encouraging them to take the patient's perspective during clinical encounters. The moderating role of individual difference factors on effectiveness of contact and student-reported hours of empathy training on patient-specific attitudes was assessed. A total of 3,576 students enrolled across 49 US medical schools completed an online survey at the start of their first year of medical school and at the end of their fourth year. Favorable contact experience with higher-weight patients predicted improved attitudes toward heavier patients after 4 years of medical school, and appeared sufficient to partially offset the effects of dislike of higher-weight individuals at baseline. The impact of favorable contact on general anti-fat attitudes was less strong, highlighting the importance of using target-specific outcome measures. The positive effects of favorable contact on attitudes toward higher-weight patients did not differ based on students' baseline levels of social dominance orientation, dispositional empathy, or need for cognitive closure. In contrast, the effectiveness of training did vary by student characteristics, generally being more effective in students who were more egalitarian and empathic at baseline, with little effect, or even adverse effects in students low in these traits. Overall, however, perspective-taking training produced only small improvements in attitudes toward higher-weight patients.

## Introduction

### Anti-fat bias in healthcare

The prevalence of anti-fat bias in Western societies has been well-established, with higher-weight individuals experiencing both interpersonal and institutional stigma across many domains of daily life (Puhl and Heuer, 2009)^1^. The consequences of anti-fat attitudes are of particular importance in healthcare settings. Explicit anti-fat bias is manifested overtly, and reflects conscious negative attitudes toward higher-weight individuals. Implicit bias, in contrast, represents ones' automatic, unintended, and often unconscious attitudes or feelings about a group. Thus, even if a healthcare provider considers him or herself to be unbiased, or at least committed to providing the same quality of care to all patients, implicit attitudes may influence their behavior or judgment (for a review, see Zestcott et al., 2016). Both explicit and implicit anti-fat bias have been documented in healthcare providers (Puhl and Heuer, 2009; Sabin et al., 2012; Brown and Flint, 2013) and medical students (Miller et al., 2013; Swift et al., 2013a; Phelan et al., 2014), and are associated with widespread patient experiences of weight stigma in medical settings (Puhl and Brownell, 2006; Hatzenbuehler et al., 2009). Findings from two studies of healthcare professionals specializing in the treatment of obesity suggest that the already high levels of explicit prejudice toward higher-weight individuals increased further between 2001 and 2013 (Schwartz et al., 2003; Tomiyama et al., 2015). Implicit anti-fat attitudes improved over the same time period, but nevertheless indicated an anti-fat, pro-thin bias (Tomiyama et al., 2015).

Weight stigma among healthcare providers has serious implications for quality of care for higher-weight patients. Evidence suggests that body size is inversely associated with patients' trust in their medical provider and treatment satisfaction, and that this effect is mediated by perceived weight stigma (Balkhi et al., 2013; Gudzune et al., 2014). Higher-weight patients report feeling that their health complaints are not taken seriously, with weight loss being prescribed for any presenting condition (Aphramor, 2013), and they are more likely to avoid accessing medical care, delay, or cancel appointments (Olson et al., 1994; Drury and Louis, 2002), and less likely to take advantage of preventive healthcare options (Fontaine et al., 1998; Østbye et al., 2005; Maruthur et al., 2009). They are also more likely to engage in “doctor shopping,” reducing continuity of care, which is then associated with greater utilization of emergency department services (Gudzune et al., 2013).

As this issue has received increasing recognition among professional bodies and educators, a number of interventions designed to reduce anti-fat bias in trainee and practicing healthcare professionals have been explored. Typical interventions include lectures, reading materials, films, or other media relating to the patient experience of weight stigma, role playing, interactions with virtual or standardized patients, and manipulation of social consensus beliefs (for a review, see Alberga et al., 2016). However, the majority of interventions have been guided by attribution theory. These interventions take an educational approach emphasizing the complex causality of obesity, and aim to challenge beliefs that weight is entirely under individual control and that higher-weight individuals, are, by definition, simply lacking in willpower or desire to change. While such studies have been relatively successful at influencing knowledge about obesity causation, they have been less successful at reducing negative attitudes toward higher-weight individuals. Further, a review and meta-analysis suggested that interventions based on attribution theory tend to produce weaker effects than those based on empathy building, social consensus theory, or more complex designs (Lee et al., 2014). Additionally, many studies are characterized by weak methodologies that limit the practical utility of their findings, including lack of control groups, non-randomized participant allocation, and absence of long-term follow-up (Alberga et al., 2016), and few studies have considered the factors that influence the success or failure of their interventions (for an exception, see O'Reilly et al., 2016). Thus, there is a clear necessity for more effective interventions and a better understanding of what drives or hinders this success.

### More patients, less prejudice?

The contact hypothesis, originally put forward by Allport (1954), suggests that one method of reducing prejudice is via favorable contact with the disliked outgroup, and this theory has been validated across a wide range of situational and intergroup contexts (Pettigrew et al., 2011). A meta-analysis of potential mechanisms of this effect concluded that positive contact experiences reduced prejudice by lessening intergroup anxiety, increasing perspective taking and emotional empathy, and to a lesser extent, enhancing knowledge about the outgroup (Pettigrew and Tropp, 2008).

A recent longitudinal study by Phelan and colleagues provided support for the role of positive contact and reduced intergroup anxiety in improving explicit anti-fat attitudes among medical students (Phelan et al., 2015b). The authors analyzed changes in attitudes toward higher-weight individuals between entry to medical school and after 4 years of medical education in 1795 fourth-year medical students, and found that favorability of contact with higher-weight patients, and to a lesser extent higher-weight faculty and staff, was associated with reduced explicit anti-fat attitudes. Favorable contact with higher-weight patients was also linked to reductions in implicit anti-fat attitudes. Confidence in providing weight-loss counseling was used as a proxy measure of intergroup anxiety and was associated with a reduction in anti-fat attitudes over the 4-year training period (Phelan et al., 2015b).

However, as noted above, some evidence suggests that explicit anti-fat attitudes have increased among clinicians specializing in the treatment of higher-weight patients in the last decade (Tomiyama et al., 2015), and recent studies continue to indicate high levels of negative stereotypical beliefs and even overt stigmatizing treatment of higher-weight patients in a range of healthcare professionals (Puhl et al., 2014; Setchell et al., 2014; Bombak et al., 2016; Garcia et al., 2016). One possible explanation is that many real-world patient interactions are not favorable and thus do not lead to improved attitudes. In fact, primary health care providers presented with vignettes depicting higher-weight patients were more likely to agree that seeing the patient would feel like a waste of their time, make them like their job less, and would annoy them more than otherwise identical lower-weight patients (Hebl and Xu, 2001). In terms of medical education, it would therefore be useful to identify factors that increase the likelihood of positive contact experiences with higher-weight patients. Another possibility is that individual difference factors may moderate the effects of contact on prejudice, such that some individuals will respond better to contact experiences than others.

It is also worth noting that the majority of previous studies have utilized a measure of general anti-fat attitudes rather than exploring attitudes toward higher-weight patients specifically. While attitudes toward higher-weight individuals in general are likely to influence attitudes toward patients in a medical setting, it is context-specific attitudes that are likely to carry the most weight in clinical encounters. Additionally, it is unclear whether attitudes toward higher-weight individuals in general and toward higher-weight patients specifically would be equivalent. On the one hand, it is possible that a healthcare professional may feel more benevolent toward a treatment-seeking individual than toward a higher-weight person observed in a social context. However, weight stigma is often linked to beliefs about the controllability of weight, with failure of personal responsibility for maintaining an “acceptable” weight being associated with higher anti-fat attitudes (Crandall et al., 2001). Thus, it is also possible that the attribution of blame conferred on higher-weight individuals for their devalued status may be intensified when those individuals are demonstrably in poor health and seeking clinical care.

### Individual difference factors as moderators of contact effects

There are individual differences in wide range of prejudiced beliefs. For example, social dominance orientation (SDO), a belief system that represents a preference for a hierarchical society in which some groups are more deserving of higher status than others, has been linked to prejudicial attitudes against a range of traditionally stigmatized groups, including higher-weight individuals, as well as support for policies that maintain this systematic inequality (Sidanius and Pratto, 2011; O'Brien et al., 2013). A tendency to be biased against a range of stigmatized targets has also been linked to several personality traits, including low levels of empathy and high need for closure (Hodson and Dhont, 2015). Empathy can be operationalized as a construct comprised of two main components: First, the ability to consider things from other people's perspectives, often labeled “cognitive empathy,” and second, a more affective response to others' situation or suffering, or “emotional empathy” (Davis, 1980). Higher levels of both cognitive and emotional empathy have been linked to lower prejudicial attitudes toward a range of stigmatized groups (Bäckström and Björklund, 2007). In the context of attitudes toward higher-weight individuals, Graziano and colleagues demonstrated that empathy had a strong, significant, negative correlation with anti-fat prejudice as measured by social distancing in two large studies of undergraduate students (Graziano et al., 2007). The need for closure (NFC) is a personality trait influencing the cognitive processes involved in knowledge formation, with high NFC reflecting a motivational drive to minimize cognitive effort whilst still alighting on an answer (Webster and Kruglanski, 1994). Individuals with a high NFC will tend to “seize” on the first suitable piece of information that presents itself, and “freeze” at this point, discounting or ignoring any further relevant information that is provided that might challenge the permanence of the currently attained closure (Kruglanski and Webster, 1996). Thus, individuals high in NFC are more likely to utilize stereotypical information in judgment formation and less likely to be swayed by evidence to the contrary (Webster and Kruglanski, 1997), and NFC has been associated with a range of racial and gender prejudices (Roets and Van Hiel, 2011a,b). Indeed, such essentialist categorization has been shown to partially mediate the relationship between NFC and racial prejudice (Roets and Van Hiel, 2011b). In addition, the preference for use of the most readily available information has been shown to reduce the likelihood of perspective taking when reading about an individual with whom one does not identify, as one's own perspective is likely to be the most readily accessible, and this in turn reduces concern or compassion for that individual (Webster and Kruglanski, 1997).

One way in which these individual difference factors may impact on prejudicial attitudes is via their effect on contact experiences with members of marginalized outgroups. Studies in both restricted (UK prisons) (Hodson, 2008) and community samples (Dhont and van Hiel, 2009) have shown that SDO moderated the effect of contact on prejudicial attitudes, such that contact reduces prejudice more in individuals who are high in SDO, with the effects among individuals low in SDO being more variable. However, other studies, including one looking at obese targets, have failed to find a moderating effect of SDO (Asbrock et al., 2012, 2013; Hodson et al., 2013; for a review, see Hodson, 2011). Likewise, in a series of studies, Dhont et al. (2011) demonstrated that individuals high in NFC displayed a greater reduction in anti-immigrant sentiment in response to positive contact with them than those low in NFC. This result held across a variety of measures of intergroup attitudes, and directionality of the effect was confirmed in field studies.

To date, the contact hypothesis literature has focused predominantly on the role of state empathy as a mediator of the relationship between contact and prejudice. State empathy is generally operationalized as a context-specific attitude or behavior, for example, one's feelings toward a particular group, rather than as a stable personality trait. To our knowledge, no published studies specifically examine the role of dispositional, or trait, empathy as a moderator of the prejudice-reducing effect of contact, although there is no theoretical reason that would preclude this phenomenon. Indeed, beyond the contact hypothesis literature, empathy has been explored as a moderator of the effectiveness of prejudice-reduction strategies. For example, in a study of Spanish older adults, instructions to take the perspective of a Moroccan immigrant reduced scores on a measure of racial prejudice, but were more effective in those with initially lower levels of empathy toward the target (Álvarez Castillo et al., 2014). In contrast, in a study of US college students, trait levels of cognitive empathy did not moderate the effectiveness of a diversity awareness course on either comfort with an ethnic outgroup or willingness to act to promote diversity (Cole et al., 2011).

### Can empathy training reduce anti-fat attitudes among medical students?

Empathy has long been recognized as a critical component of good medical practice. A recent review of personality factors and outcomes in medical education and practice identified dispositional empathy as one of the most important predictors of both academic and professional achievement during medical training (Hojat et al., 2013). As a result, the Association of American Medical Colleges recommends that medical education include a focus on empathy (Association of American Medical Colleges, 1999), and the vast majority of medical schools include curricula on specific techniques designed to promote empathic practice.

In the previously described study of factors influencing anti-fat attitudes during medical school, Phelan and colleagues also tested the effect of a composite measure of interpersonal skills training, which included training the students had received in taking the patient's perspective. They reported that the number of hours of training in interpersonal skills received did not significantly predict changes in either explicit or implicit anti-fat attitudes among medical students (Phelan et al., 2015b). However, several types of interpersonal skills were combined in this measure; as well as perspective taking, it included communication skills in general, partnership building skills, and working effectively in inter-professional teams. Thus, the relative contribution of perspective-taking skills training to this measure would likely be highly variable, and it may be informative to explore the unique impact of training in perspective taking in influencing anti-fat attitudes.

### Individual difference factors as moderators of contact effects

As with patient contact experiences, it is also possible that differences in personality or belief systems may result in empathy skills training being more effective in some students than in others, depending on individual characteristics. A longitudinal evaluation of diversity training effectiveness in a general student sample found that dispositional empathy moderated the effect of perspective-taking training on attitudes and supportive behaviors toward gay and African American individuals, such that the training only benefitted students who were low in dispositional empathy prior to the intervention (Lindsey et al., 2015). However, to our knowledge, the moderating role of participant characteristics on training effectiveness has not been tested in the context of medical education.

### The present study

The aims of the present study were three-fold. First, we aimed to extend the findings of Phelan et al. (2015b) regarding the relationship between contact and anti-fat attitudes in general to the effect of attitudes toward higher-weight patients specifically. To this end, we explored the predictive role of demographics, BMI, anti-fat attitudes at the start of medical training, and contact experiences with higher-weight individuals both before and during medical school on final-year measures of both general anti-fat attitudes and negative attitudes toward higher-weight patients. Consistent with the wider literature we expected age, female gender, and higher BMI to be associated with lower anti-fat attitudes. We also predicted that positive contact experiences with higher-weight individuals, especially patients, would improve both anti-fat attitudes in general and attitudes toward higher-weight patients specifically, but we made no *a priori* predictions about the relative strength of the associations for patient-directed vs. general anti-fat attitudes.

A second aim was to identify which individual and situational factors affecting medical students predicted their reported favorability of contact with higher-weight patients. If, as expected, positive contact experiences were associated with reduced prejudice, future medical training might benefit from a focus on increasing positive contact experiences with higher-weight patients. Thus, it would be helpful to know what factors either increase or decrease the likelihood of rating contact with higher-weight patients as being positive. In addition to the variables explored above, whose effects on positive ratings were hypothesized to be consistent with their impact on prejudicial attitudes, the predictive role of a number of individual difference measures was assessed. We expected that NFC and SDO would reduce the likelihood of students classifying their interactions with higher-weight patients as being positive, and that dispositional empathy would increase the likelihood of reporting favorable interactions.

Finally, we examined whether individual difference variables moderated the relationship between medical school experiences and attitudes toward higher-weight patients. We focused on two aspects of the medical school experience—contact with higher-weight patients and training designed to increase perspective taking in the doctor-patient encounter. We focused on contact with higher-weight patients only, rather than including contact with higher-weight peers or hospital faculty, as we expected that contact with higher-weight patients would be both more frequent than contact with other higher-weight individuals in the medical school environment, and more influential in terms of affecting patient-directed attitudes.

Likewise, although medical schools now routinely offer at least some training in a range of interpersonal skills aimed at improving the doctor-patient relationship and standards of clinical care, based on the extant literature regarding the role of perspective taking in prejudice reduction, we predicted that training designed to increase empathy would likely have the greatest impact on reducing prejudicial attitudes toward this stigmatized patient group. The purpose of exploring the moderating role of individual difference variables in these prejudice-reduction pathways was to ascertain whether contact or training effects are stronger in some students than in others. If the anti-fat attitudes of some students are more or less responsive to medical school experiences, being able to identify these students at intake may facilitate improved selection and/or better-targeted training. Based on the largely consistent findings cited above, we expected that the positive impact of both contact and training on attitudes toward higher-weight patients would be greatest in students who were higher in SDO and NFC and lower in dispositional empathy at the start of their medical training.

## Methods

### Sample

The sample was part of the longitudinal Medical Student Cognitive Habits and Growth Evaluation Study (CHANGES) (NHLBI R01HL085631, PI van Ryn). Details of the cohort and data collection methods have been described previously (van Ryn et al., 2014). Briefly, a variety of methods were used to contact students at a random selection of 50 medical schools from strata of public and private schools in six regions of the United States. One of the sampled schools, a military school, was atypical compared with the rest of the sample and their data were excluded from analyses. Eligible students were invited to complete an online survey during their first semester of their first year of medical school in fall of 2010. Responses were examined for indications of inattentive responding (e.g., implausibly short completion times, systematic repetition of identical scoring choices). After removal of invalid and duplicate surveys (*n* = 32), a total of 4,732 students from 49 schools provided completed questionnaires at this time. Respondents completing surveys at baseline were invited to complete a second online questionnaire during their final semester in spring of 2014, with 3,756 (79%) completing the follow-up survey. In the present analysis, only participants with data at both baseline and year 4 were included. A sub-sample of 50% of the cohort completed a Weight Implicit Association Test (Weight IAT) to measure implicit anti-fat bias; the remainder of the students completed a sexual orientation IAT. Implicit anti-fat bias data were available for 1,795 students in the final sample. Students completing the follow-up survey at year 4 did not differ from non-completers on demographic variables, BMI, social desirability responding, contact with higher-weight individuals prior to medical school, baseline explicit or implicit anti-fat attitudes, emotional or cognitive empathy, “seizing” tendencies, or egalitarianism. However, completers scored slightly lower on baseline “freezing,” and elitism^2^. This study was approved by the Institutional Review Boards of the Mayo Clinic and the University of Minnesota. At both time points, respondents gave informed consent and received a $50.00 incentive.

### Measures

Students' age, gender, race, and annual family income were measured with standard survey questions at baseline. For the present analyses, race was dichotomized into Non-Hispanic White vs. all other races. Students also self-reported height and weight, which were used to calculate body mass index (BMI).

As this study was part of a larger survey, a number of steps were taken to minimize participant burden. First, constructs were tested using a reduced number of items from validated scales, with included items selected on the basis of construct and face validity, and with input from scale authors where possible. Secondly, all measures were scored from 1 (“Strongly disagree”) to 7 (“Strongly agree”) unless otherwise stated.

Anti-fat attitudes were measured with items selected from the Anti-Fat Attitudes Questionnaire (AFAQ; Crandall, 1994). Items made up three subscales: “Dislike,” “Fear of Fat,” and “Willpower,” with higher scores indicating more negative attitudes. In the present analyses, baseline scores on the three subscales of the AFAQ were used as predictors of year-4 general and patient-specific anti-fat attitudes and of reported future contact favorability. Cronbach's alpha was 0.85 for Dislike, and Spearman-Brown coefficients were 0.79 for Fear of Fat, and 0.73 for Willpower at baseline. Scores obtained on the Dislike subscale during the follow-up survey were used as the measure of general anti-fat attitudes post-medical school. Cronbach's alpha for this subscale was 0.84 in year 4.

Negative attitudes toward higher-weight patients were measured using five items from the Attitudes about Treating Obese Patients scale (Puhl et al., 2014): I dislike treating obese patients; Treating obese patients is professionally rewarding (reverse scored); I feel more irritated when I am treating an obese patient than when I am treating a non-obese patient; I feel disgust when treating an obese patient; It is difficult to feel empathy for an obese patient. Cronbach's alpha for this measure was 0.87 in the present sample. Higher scores indicate more prejudicial attitudes. This measure was assessed at year 4 only.

Implicit weight bias at baseline was used as a predictor of year-4 general and patient-specific anti-fat attitudes and of reported future contact favorability. Implicit weight bias was measured using the Weight IAT. The IAT has been extensively validated as a measure of unconscious attitudes and has good predictive validity for prejudicial behavior independent of explicit attitudes (Hofmann et al., 2005). The Weight IAT is a computerized task in which participants have to categorize silhouettes of fat or thin bodies with either positive or negative words. If an individual attributes negative stereotypes to a group, in this case high-weight individuals, it takes them longer to correctly categorize the image with a positive word than with a negative word, and this time differential over repeated trials is an indicator of the extent of unconscious negative biases against the group (Greenwald et al., 2003, 2009; Nosek et al., 2007). Difference scores are calculated for fat-bad/thin-good pairings vs. fat-good/thin-bad pairings and can range from –2, indicating a strong pro-fat bias, to +2, indicating a strong pro-thin bias. Thus, higher (more positive) scores, indicate an implicit preference for thin over fat bodies.

Frequency and favorability of contact with obese individuals were measured for four separate target groups: Contact with obese people prior to medical school was assessed at baseline, and contact with obese medical students, patients, and faculty or staff during medical school was assessed during the follow-up survey. Four-point Likert scales were used to register the frequency (1 = “None,” 2 = “Little,” 3 = “Some,” 4 = “Substantial”) and favorability (1 = “Very unfavorable,” 2 = “Unfavorable,” 3 = “Favorable,” 4 = “Very favorable”) of contact.

Perspective-taking skills training was assessed with a single item about the number of hours of training students had received pertaining to awareness and consideration of the patient's perspective during clinical encounters. This item was part of a broader series of questions on communication skills training during medical school, but only the item regarding perspective taking was included in the present analysis. The following wording was used: “In the past 4 years, about how many training hours did your medical school provide on seeing things from your patient's perspective (could have been called perspective taking or patient-centeredness)?” Participants were informed that “training hours” might include such activities as lectures, learning activities, small group work sessions, demonstrations, and evaluations specifically focused on a topic or skill set. Responses were indicated on a sliding scale including whole numbers between 0 and 49 h, and anchored at the upper end with “50 or more hours”.

Individual difference measures were collected for two purposes. Baseline scores of SDO, NFC, and cognitive and emotional empathy were explored as potential predictors of positive contact experience and as moderators of the effectiveness of contact and perspective-taking skills training.

Social dominance orientation was measured with three items each from the Egalitarianism and Elitism subscales of the Social Dominance Orientation scale (Pratto et al., 1994). The Egalitarianism subscale includes items such as, “We would have fewer problems if we treated different groups more equally” and the Elitism subscale includes items such as, “It's probably a good thing that certain groups are at the top and some at the bottom.” In the present sample, Cronbach's alphas were 0.83 and 0.87 for the Egalitarianism and Elitism subscales, respectively.

Need for cognitive closure was measured using items from three subscales of the Need For Closure scale (Webster and Kruglanski, 1994). The Discomfort with Ambiguity and Preference for Predictability subscales were used to capture tendency to experience discomfort or distress at uncertain knowledge or situations, or “seizing,” whereas the Closed-mindedness subscale was used to capture a preference for stable knowledge and unwillingness to challenge current knowledge, or “freezing.” Consistent with previous work (van Hiel et al., 2004), principal components analysis with direct oblimin rotation confirmed that these subscales loaded onto the two hypothesized factors. Several items had low factor loadings and reduced scale reliabilities. After their removal, the first factor, representing distress at uncertain knowledge, comprised 9 items and had good internal reliability (Cronbach's alpha 0.81). The second factor, representing preference for stable knowledge, comprised seven items and had acceptable reliability (Cronbach's alpha 0.64).

Cognitive and emotional empathy were measured with the Perspective Taking and Empathic Concern subscales, respectively, of the Interpersonal Reactivity Index (Davis, 1983). The Perspective Taking subscale included five items, such as, “I try to look at everybody's side of a disagreement before I make a decision,” and the Empathic Concern subscale included seven items, such as, “When I see someone being taken advantage of, I feel kind of protective toward them.” Cronbach's alpha was 0.75 for Perspective Taking and 0.83 for Empathic Concern.

Finally, the tendency to respond to questions in a “socially acceptable” manner was assessed using seven items from the Marlowe-Crowne Social Desirability Scale (Crowne and Marlowe, 1960). Cronbach's alpha in the present sample was 0.63.

### Data analysis

Descriptive statistics were calculated for sample characteristics and anti-fat attitudes. Bivariate correlations were calculated between baseline characteristics and general and patient-specific anti-fat attitudes at year 4. Partial correlations controlling for social desirability responding were also calculated.

#### Predictors of general and patient-specific anti-fat attitudes

Hierarchical linear regression models were used to explore predictors of dislike of heavier individuals in general and negative attitudes toward heavier patients in particular as measured at year 4. In the models exploring the role of contact, demographic variables, BMI, baseline general anti-fat attitudes, and pre-medical school contact experiences with higher-weight individuals were added in step 1. Medical school contact experiences with higher-weight peers, faculty and staff, and patients were added at step 2. In the models exploring the role of empathy skills training, only demographic variables, BMI, and baseline anti-fat attitudes were added at step 1, with hours of perspective-skills training added at step 2. As the presence of heteroscedasticity may have a notable impact on inferential statistics in large samples, regressions were performed using the HCREG macro for SPSS (Hayes and Cai, 2007) to calculate heteroscedasticity-consistent standard errors.

#### Predictors of positive contact experiences

A series of logistic regressions were conducted to identify the impact on reported contact favorability of (i) demographic and anthropometric variables, (ii) general anti-fat attitudes, (iii) individual difference factors, and (iv) favorability and frequency of contact with higher-weight individuals before and during medical school. With the exception of medical school contact experiences, baseline scores were used for all variables as predictors of future attitudes. The dependent variable was respondents' ratings of positive contact experiences with higher-weight patients. Most contact experiences were reported to be either “Favorable” or “Very favourable,” with “Very unfavorable” encounters being relatively infrequent. As the overall valence was deemed to be the most important distinction, the dependent variable was dichotomized into “Positive” (“Favorable” or “Very favorable”) and “Negative” (“Unfavorable” or “Very unfavorable”) for the purpose of the logistic regression analyses.

#### Moderation of contact and training on attitudes toward higher-weight patients by differences in individual characteristics

Moderation effects were tested in a series of simple moderation models using PROCESS (Hayes, 2013) for SPSS model 1. Either favorability of contact with higher-weight patients or hours of training in taking the patient's perspective were used as predictors of negative attitudes toward higher-weight patients. Potential moderators were tested individually, and variables were mean-centered prior to calculation of interaction terms. Age, race, gender, and BMI were included as covariates in all models. As individual difference measures were somewhat non-normal, with means toward the more “ideal” end of each scale and small standard deviations, interaction effects were probed across the range of values, at the 10th, 25th, 50th, 75th, and 90th percentile. The interaction effects were visualized using simple slopes plotted with low and high values of the predictor and moderator at the 10th and 90th percentile, respectively.

## Results

Sample characteristics are shown in Table 1. The baseline demographic characteristics of the sample are similar to those of all students matriculating at U.S. medical schools in the same year (Association of American Medical Colleges, 2011). Socioeconomic data are consistent with prior data on parental income (Association of American Medical Colleges, 2008). Sample sizes vary due to missing data; percentages reported are of the non-missing cases for each variable.

**Table 1:** **Sample characteristics**.

	**Mean**	***SD***	**Actual range**	***N***
**DEMOGRAPHICS**				
Age	23.9	2.6	19–49	3,727
Gender				3,756
Male	49.9%			
Female	50.1%			
Race				3,756
White	71.6%			
Other	28.4%			
Family income				3,485
Below $20,000	4.5%			
$20,000–49,999	10.7%			
$50,000–99,999	23.4%			
$100,000–249,999	41.1%			
Over $250,000	20.2%			
BMI	23.2	3.5	15.0–48.9	3,739
Underweight (BMI <18.5)	3.6%			
Normal weight (BMI 18.5–24.9)	72.5%			
Overweight (BMI 25.0–29.9)	19.4%			
Obese (BMI > 30.0)	4.5%			
**ANTI-FAT ATTITUDES**				
**Y1**				
AFAQ–Dislike	2.3	1.4	1–7	3,716
AFAQ–Fear of fat	4.5	1.8	1–7	3,716
AFAQ–Willpower	4.0	1.5	1–7	3,708
Implicit association test^a^	0.42	0.44	–1.5 to 1.5	1,887
**Y4**				
AFAQ-Dislike	2.5	1.5	1–7	3,727
AFAQ–Fear of fat	4.8	1.7	1–7	3,728
AFAQ–Willpower	4.0	1.6	1–7	3,727
Implicit association test^a^	0.31	0.42	–1.8 to 1.4	1,838
Negative attitudes toward obese patients	3.3	1.2	1–7	3,690
**CONTACT WITH HIGHER-WEIGHT INDIVIDUALS**				
**Quantity^b^**				
Before medical school	2.9	0.8	1–4	3,691
Obese staff, faculty, interns	2.6	0.7	1–4	3,680
Obese medical students	2.4	0.7	1–4	3,680
Obese patients	3.8	0.5	1–4	3,680
**Favorability^b^**				
Before medical school	3.2	0.6	1–4	3,672
Obese staff, faculty, interns	3.4	0.6	1–4	3,625
Obese medical students	3.4	0.6	1–4	3,633
Obese patients	3.2	0.7	1–4	3,652
Positive^c^	86.9%			
Negative^d^	13.1%			
**Y1 INDIVIDUAL DIFFERENCE MEASURES**				
Elitism	1.8	1.1	1–7	3,697
Egalitarianism	5.1	1.3	1–7	3,699
Cognitive empathy	5.3	0.9	1–7	3,683
Emotional empathy	5.6	0.9	2.1–7.0	3,682
Need for closure—Seizing	4.5	0.9	1.2–6.9	3,705
Need for closure—Freezing	3.0	0.7	1–7	3,705
Hours training in perspective-taking skills	21.1	15.4	0–50+	3,453

*All questionnaire measures had a possible range of 1–7 unless otherwise noted*.

a Possible range –2 to +2;

b Possible range 1 to 4;

c Overall contact with higher-weight patients rated as either “Favorable” or “Very favorable”;

d Overall contact with higher-weight patients rated as either “Unfavorable” or “Very unfavorable.”

Scores on SDO, cognitive and emotional empathy, explicit measures of prejudice, and favorability of contact were skewed toward the more “desirable” end of the scales, whereas implicit anti-fat attitudes indicated a moderate preference for thin people over fat people.

Bivariate correlations indicated small to moderate associations between social desirability responding and measures of explicit anti-fat attitudes, reported favorability of contact with higher-weight individuals, and scores on individual difference measures^3^. However, partial correlations controlling for social desirability responding did not significantly alter the relationships between the remaining variables.

### Predictors of general and patient-specific anti-fat attitudes

#### Contact

Table 2 shows the results of hierarchical linear regressions exploring the role of medical school contact with higher-weight individuals as predictors of general and patient-specific anti-fat attitudes at year 4 whilst controlling for baseline anti-fat attitudes and contact experience prior to entering medical school. Demographic variables, BMI, baseline general anti-fat attitudes, and pre-medical school contact experiences with higher-weight individuals were added in step 1. Medical school contact experiences with higher-weight peers, faculty and staff, and patients were added at step 2. Note, in the full model, baseline IAT was not a significant predictor of either year 4 anti-fat attitudes or attitudes toward higher-weight patients. As the IAT-Weight was completed by only half of the sample, data are presented here for analyses without inclusion of baseline IAT scores.

**Table 2:** **Linear regression models showing predictors of general and patient-specific anti-fat attitudes**.

**Y4 AFAQ dislike**									**Y4 negative attitudes to obese patients**							
	**Model 1**				**Model 2**				**Model 1**				**Model 2**			
	***B***	***SE***	**β**	***p***	***B***	***SE***	**β**	***p***	***B***	***SE***	**β**	***p***	***B***	***SE***	**β**	***p***
**DEMOGRAPHICS**																
Age	–0.04	0.01	–**0.06**	**0.00**	–0.04	0.01	–**0.06**	**0.00**	–0.03	0.01	–**0.06**	**0.00**	–0.03	0.01	–**0.06**	**0.00**
Gender	0.16	0.05	**0.05**	**0.00**	0.14	0.05	**0.05**	**0.00**	0.27	0.04	**0.11**	**0.00**	0.26	0.04	**0.11**	**0.00**
Race	–0.05	0.05	–0.01	0.32	–0.05	0.04	–0.02	0.26	0.05	0.04	0.02	0.19	0.03	0.04	0.01	0.35
BMI	–0.01	0.01	–**0.03**	**0.04**	–0.01	0.01	–0.03	0.07	–0.03	0.01	–**0.08**	**0.00**	–0.03	0.01	–**0.07**	**0.00**
**Y1 ANTI-FAT ATTITUDES**																
Dislike	0.48	0.02	**0.46**	**0.00**	0.45	0.02	**0.43**	**0.00**	0.26	0.02	**0.30**	**0.00**	0.23	0.02	**0.26**	**0.00**
Fear	0.05	0.01	**0.05**	**0.00**	0.04	0.01	**0.05**	**0.00**	0.06	0.01	**0.08**	**0.00**	0.05	0.01	**0.07**	**0.00**
Willpower	0.10	0.02	**0.10**	**0.00**	0.09	0.02	**0.09**	**0.00**	0.13	0.01	**0.17**	**0.00**	0.12	0.01	**0.15**	**0.00**
**PRE-MED CONTACT**																
Frequency	–0.05	0.03	–0.02	0.06	–0.02	0.03	–0.01	0.45	–0.09	0.03	–**0.06**	**0.00**	–0.05	0.02	–**0.03**	**0.04**
Favorability	–0.19	0.04	–**0.08**	**0.00**	–0.07	0.04	–0.03	0.06	–0.20	0.03	–**0.10**	**0.00**	–0.06	0.03	–**0.03**	**0.04**
**MEDICAL SCHOOL CONTACT**																
**Frequency of Contact**																
Obese peers					0.04	0.04	0.02	0.29					–0.02	0.03	–0.01	0.62
Obese staff, faculty, interns					–0.08	0.04	–**0.04**	**0.04**					–0.05	0.03	–0.03	0.10
Obese patients					–0.07	0.04	–0.03	0.07					–0.06	0.03	–0.03	0.07
**Favorability of Contact**																
Obese peers					–0.13	0.06	–**0.05**	**0.04**					0.04	0.05	0.02	0.47
Obese staff, faculty, interns					0.03	0.06	0.01	0.60					0.03	0.05	0.02	0.54
Obese patients					–0.46	0.04	–**0.21**	**0.00**					–0.62	0.04	–**0.34**	**0.00**
*R*^2^ change	0.34 (*p* = 0.05)								0.26 (*p* = 0.11)							
*R*^2^ full model	0.39								0.37							

*Gender: 1 = Male, 0 = Female; Race: 1 = White, 0 = Other. B, unstandardized regression coefficient; β, standardized regression coefficient. Bold font indicates significant predictors at p < 0.05 level*.

The full models predicted a significant and similar proportion of the variance in both general anti-fat attitudes (*R*^2^ = 0.39) and negative attitudes toward obese patients (*R*^2^ = 0.37, both *p* < 0.001). This is perhaps unsurprising, as general and patient-specific anti-fat attitudes were highly correlated (Spearman's rho = 0.70, *p* < 0.001). Baseline dislike of higher-weight individuals was the strongest predictor of dislike at year 4, and this was only partially offset by favorable contact with higher-weight patients. Positive contact with higher-weight peers and frequent contact with higher-weight faculty were associated with small improvements in general anti-fat attitudes. Interestingly, positive contact experience prior to medical school became non-significant when medical school experiences were added into the model.

In contrast, while baseline dislike of higher-weight individuals was a major and significant predictor of attitudes toward higher-weight patients at year 4, the contribution to variance explained was smaller than was the case for general anti-fat attitudes, and positive contact experiences with higher-weight patients had a much larger and inverse relationship with negative attitudes toward such patients. Overall, medical school contact experiences accounted for 5.3% additional variance in anti-fat attitudes in general, but twice that for patient-specific attitudes (Z = 4.60, 1-tailed *p* < 0.001). More frequent and more favorable contact with higher-weight individuals prior to medical school also remained significantly associated with better attitudes toward higher-weight patients, even when medical school experiences were included in the model; however, contact with higher-weight peers and faculty had no significant impact on patient-directed attitudes. Weight controllability beliefs—that is, the extent to which the students believed that higher-weight status was due to lack of willpower, was also a significant predictor of more negative attitudes, both general and patient-specific, and fear of fat also had a small but significant effect. Male gender and younger age were associated with more negative attitudes. In addition, higher BMI was associated with less negative attitudes toward higher-weight patients, but BMI was not a significant predictor of anti-fat attitudes in general.

Multicollinearity diagnostics for all regressions identified dependency between favorability of contact with higher-weight peers and higher-weight faculty and staff [both variance inflation factors (VIFs) = 3.4, 80 and 72% of the variance of the respective regression coefficients associated with the smallest eigenvalue of 0.005; all other VIFs ≤ 2.0], and these measures were highly correlated (*r* = 0.82). However, positive contact with higher-weight faculty and peers was not a significant predictor of attitudes toward higher-weight patients, and the two constructs differentially predicted general anti-fat attitudes. Additionally, standard errors were not inflated, and the large sample size may have reduced the impact of this dependency. Thus, multicollinearity appears not to have been a limiting issue in these analyses.

#### Perspective-taking skills training

Hierarchical linear regressions were used to explore the role of perspective-taking skills training during medical school on general and patient-specific anti-fat attitudes at year 4. Demographic variables, BMI, baseline general anti-fat attitudes were added in step 1. Hours of skills training in perspective taking were added at step 2. Hours of skills training did not significantly explain additional variance in either general (*R*^2^ change = 0, *p* = 0.47) or patient-specific anti-fat attitudes (*R*^2^ change = 0.002, *p* = 0.07) at year 4, when controlling for baseline attitudes.

### Predictors of reporting favorable contact with obese patients

Table 3 summarizes the results of the logistic regression analyses assessing the impact of a range of variable types on the likelihood of students classifying their contact experiences with obese patients as being positive. Again, multicollinearity diagnostics identified shared variance between favorability of contact with obese peers and obese faculty and staff (both VIFs = 3.1, 89 and 91%, respectively, variance associated with an eigenvalue of 0.005; all other VIFs ≤ 1.8). No inflation of standard errors was evident. The strongest predictors for reporting positive contact experiences were favorability of contact with other obese individuals. Students reporting positive contact with higher-weight peers, faculty and staff or obese individuals before entering medical school were twice as likely (ORs 1.95–2.57, *p* < 0.001) to report that their contact with obese patients was positive (see the discussion for implications of dependency of contact variables). Emotional empathy was also associated with a 23% higher likelihood of reporting favorable contact with obese patients (OR 1.23, *p* < 0.01). Baseline explicit and implicit anti-fat attitudes and weight controllability beliefs, although not Fear of Fat, were strong negative predictors of reporting favorable contact experiences, with implicit attitudes having the strongest negative association.

**Table 3:** **Logistic regression analyses showing predictors of classifying contact with higher-weight patients as positive**.

	**Odds ratio**	***p***
**DEMOGRAPHIC AND ANTHROPOMETRIC VARIABLES**		
Gender (1 = Male, 0 = Female)	0.97	0.76
Age	1.03	0.13
Race (1 = White, 0 = Other)	0.82	0.06
BMI	1.03	0.05
**Y1 ANTI-FAT ATTITUDES**		
AFAQ–Dislike	**0.78**	**0.00**
AFAQ–Fear of Fat	0.93	0.11
AFAQ–Willpower	**0.81**	**0.00**
Weight IAT	**0.62**	**0.00**
**Y1 INDIVIDUAL DIFFERENCE VARIABLES**		
Elitism	0.97	0.54
Egalitarianism	1.08	0.08
Cognitive empathy	1.10	0.17
Emotional empathy	**1.23**	**0.00**
Need for closure—Seizing	**0.89**	**0.04**
Need for closure—Freezing	0.89	0.20
**PRE-MED CONTACT**		
Frequency of contact	1.10	0.19
Favorability of contact	**1.95**	**0.00**
**MEDICAL SCHOOL CONTACT**		
**Frequency of Contact**		
Obese peers	1.07	0.50
Obese staff, faculty, interns	1.12	0.29
Obese patients	**0.62**	**0.00**
**Favorability of Contact**		
Obese peers	**2.57**	**0.00**
Obese staff, faculty, interns	**2.14**	**0.00**

*Bold font indicates significant predictors at p < 0.05 level*.

*AFAQ, Anti-Fat Attitudes Questionnaire; IAT, Implicit Association Test*.

Students who reported more frequent contact with higher-weight patients were significantly less likely to report that this contact was positive; however, frequency of contact with other higher-weight individuals before or during medical school did not impact on patient contact favorability ratings. Discomfort with ambiguity and unpredictability (“seizing”) was associated with an approximately 10% lower likelihood of reporting positive contact experiences (OR 0.89, *p* = 0.04), however no effects were seen for SDO, perspective taking, discomfort with unstable knowledge (“freezing”), BMI, or demographic variables. Demographic factors and BMI did not significantly increase or decrease the likelihood of reporting positive contact.

### Moderators of contact and training effects

Favorable contact with higher-weight patients was associated with similar reductions in negative attitudes, irrespective of individual levels of SDO, cognitive or emotional empathy, or NFC—that is, there was no interaction effect between contact and any of the personality or belief subscales. Further, the conditional effect of contact on attitudes toward higher-weight patients was significant at all values of the individual difference variables: However high or low an individual scored on SDO, emotional empathy, perspective-taking, or NFC, positive contact had a consistent and significant positive impact on attitudes toward higher-weight patients.

**Figure 1:**
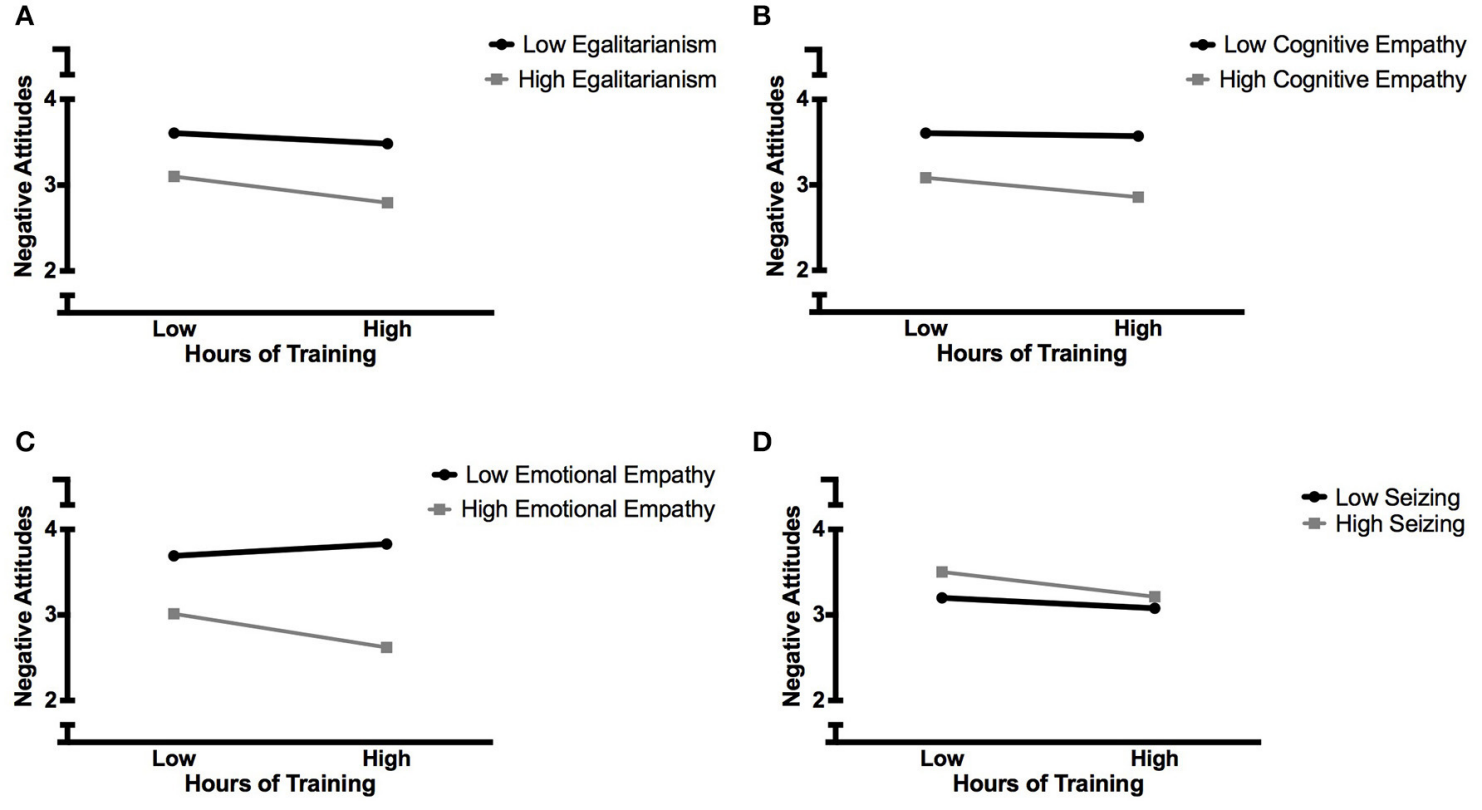
**Individual differences moderate the impact of training in perspective-taking skills on negative attitudes toward higher-weight patients**. **(A)** Egalitarianism; **(B)** Cognitive empathy; **(C)** Emotional empathy; **(D)** Need for closure—seizing. Negative Attitudes scored 1–7.

Training in perspective taking was associated with a small improvement in attitudes toward higher-weight patients—approximately 1.7% per 10 h of training, controlling for age, gender, race, and BMI. However, individual characteristics moderated the impact of training in perspective taking on attitudes toward heavier patients. Baseline levels of elitism had little impact on training effectiveness for prejudice reduction, with similar decreases observed at high and low levels of elitism; however, contrary to predictions, a slightly pronounced effect was observed for students with higher levels of egalitarianism at baseline (1.2% improvement per 10 h of training, *p* < 0.01; Figure 1A); no significant improvement was observed for students low in egalitarianism at baseline.

Likewise, while training resulted in small improvements in attitudes for students with high levels of cognitive (0.8% per 10 h, *p* = 0.03) and emotional (1.3% per 10 h, *p* < 0.001) empathy at baseline, it had no effect at all in students who were low in cognitive empathy at baseline (Figure 1B) and a small, but not statistically significant, adverse effect in students who were low in emotional empathy at baseline (Figure 1C).

Training in perspective taking was associated with similar reductions in prejudicial attitudes toward higher-weight patients in students with high and low need for stable knowledge (“freezing”), but differences were observed for students who varied in comfort with uncertain knowledge (“seizing”); in line with our hypotheses, attitudes toward higher-weight patients improved by 1% per 10 h of training (*p* < 0.01) in those who were high on this characteristic at baseline (Figure 1D).

## Discussion

The present study is the first to examine the relationship between medical school experiences and students' attitudes toward higher-weight patients specifically, rather than their anti-fat attitudes in general. While the two constructs are highly related, they are nevertheless non-equivalent, and there were some differences in predictors of the two outcomes. Thus, it may be important for future studies of attitudes in healthcare professionals to evaluate patient-specific attitudes rather than general anti-fat attitudes. Consistent with the contact hypothesis, favorable interactions with higher-weight patients were significantly associated with less negative attitudes toward them. Encouragingly, favorable contact with higher-weight patients in medical school appeared sufficient to offset the impact of year 1 anti-fat attitudes on patient-specific attitudes at year 4; even so, baseline explicit anti-fat attitudes and greater belief in the controllability of weight were still significant predictors of negative attitudes toward higher-weight patients. Further, while dislike of higher-weight individuals in general was a statistically significant predictor of negative attitudes toward obese patients, its contribution was smaller in the patient-specific measure than in the general measure. Thus, it is possible that general dislike against higher weight people may be tempered somewhat in clinical settings, although this should be tested using more objective methods. Baseline implicit anti-fat attitudes did not significantly predict either general or patient-specific explicit attitudes at year 4. This is consistent with previous findings that explicit and implicit anti-fat attitudes are only weakly related (Nosek et al., 2007; Sabin et al., 2012; Phelan et al., 2014), and may manifest differently in clinical encounters (Phelan et al., 2015a; Zestcott et al., 2016). Implicit attitudes more strongly influence non-verbal communication and stereotype utilization when under stress, whereas explicit attitudes are more closely related to overt communication and conscious decision-making, and both have implications for the development and maintenance of healthcare disparities (Dovidio et al., 1997; van Ryn and Fu, 2003; van Ryn et al., 2011).

The present analysis also revealed that medical students' individual characteristics predicted the degree to which they reported positive contact with higher-weight patients. In particular, higher levels of emotional empathy were associated with a greater tendency to categorize interactions as being favorable. In contrast, greater discomfort with uncertain knowledge, or the need to “seize” on first impressions, was associated with a reduced likelihood of rating such meetings favorably. Unsurprisingly, baseline explicit and implicit anti-fat attitudes, and greater belief in the controllability of weight, were associated with lower likelihood of rating contact with higher-weight patients favorably. In particular, a 1-point increase in implicit attitude score was associated with nearly a 40% reduction in the likelihood that interactions with heavier patients were viewed in a positive light. Few interventions have been successful at improving implicit weight bias (Daníelsdóttir et al., 2010; Swift et al., 2013b), although analysis of the present dataset by Phelan and colleagues found that favorable contact with higher-weight patients (but not higher-weight peers or clinical staff) was associated with improvements in weight IAT scores between Y1 and Y4 (Phelan et al., 2015b). Unfortunately, frequency of interactions with higher-weight patients was associated with reporting that such interactions were unfavorable. It is possible that a high frequency of heavier individuals presenting in a clinical setting serves to consolidate negative weight-based stereotypes, and, coupled with beliefs about the controllability of weight, may intensify dislike and reduce the likelihood of remembering these interactions in a positive light. This is supported by the fact that *frequency* of contact with higher-weight peers and clinical staff was not associated with favorability of contact with higher-weight patients. This contrasts with the impact of *favorability* of contact with non-patients, whereby positive contact experiences with higher-weight peers and clinical staff did significantly predict a greater likelihood of reporting contact with higher-weight patients as being positive. It should be noted, however, that the strong association between reporting favorable contact with higher-weight patients and higher-weight others may reflect a more open or agreeable temperament in general, as much as it is suggestive of student-patient interactions being influenced by student-other interactions. Indeed, the observed collinearity between reported favorability of contact with higher-weight peers and higher-weight faculty and staff would be consistent with a common underlying cause. These alternative possibilities cannot be definitively tested with the present data; however, it is likely that both mechanisms are important. For example, Jackson and colleagues have demonstrated that openness and agreeableness lead to more favorable intergroup attitudes, in part, because they increase the probability of positive intergroup contact (Jackson and Poulsen, 2005; Jackson et al., 2016). Likewise, the extensive meta-analysis of research on the contact hypothesis conducted by Pettigrew and Tropp found significant evidence that the positive impact of contact on prejudicial attitudes generalized to the entire outgroup, and across situations (Pettigrew and Tropp, 2006), making it likely that positive contact with higher-weight peers and clinical staff would also improve attitudes toward higher-weight patients.

Contrary to hypotheses, the positive effect of favorable contact on attitudes appeared to be independent of individual characteristics. It is possible that this is due to low variation on these characteristics in the present sample, with most students scoring at the more “desirable” end of each measure. In contrast, individual characteristics did moderate the association between perspective-taking training and more positive attitudes toward higher-weight patients. Although the impact of such training was small, it was nevertheless associated with improvements in attitudes toward higher-weight patients, with the greatest effects among students who had higher “seizing” scores at baseline. As noted above, individuals prone to “seizing” are more likely to rely on stereotypical judgments and essentialist categorization of a target group (Webster and Kruglanski, 1997; Roets and Van Hiel, 2011b), and in the present sample, “seizing” was also associated with a reduced likelihood of rating contact with higher-weight patients in a positive manner. Thus, this finding is encouraging in that greater benefits were observed in students who may have engaged in less perspective taking at baseline. Given that the number of hours of training was typically low, that the content and quality of such training would likely vary considerably between schools, and the fact that it was unlikely to have been specific to heavier patients, even minor improvements should be welcomed. Findings from a number of experimental studies have suggested that individuals who are high in NFC may be more sensitive to evidence suggesting that their pre-existing knowledge is inaccurate, particularly when their self-image is under threat, resulting in reduced reliance on stereotypical information in attitude formation (Kruglanski et al., 2012; Kossowska et al., 2016). Thus, one practical approach in terms of medical education may be to ensure that students are exposed to counter-stereotypical images, knowledge, and experiences with higher-weight patients, combined with psychoeducation regarding the impact of NFC on reliance on stereotypes, and reinforcement of students' motivations to act as moral individuals. However, this has yet to be tested empirically.

By contrast, the fact that training in perspective taking was more successful at improving attitudes toward higher-weight patients in students who were high in egalitarianism and both cognitive and emotional empathy at baseline, and had a small adverse effect in those with low scores on emotional empathy at baseline, may suggest that such training is largely preaching to the converted, and there is some suggestion that interventions designed to increase empathy may have antithetical effects (Stephan and Finlay, 1999; Vorauer et al., 2009). In a systematic review of studies of anti-fat attitude reduction interventions, strategies designed to evoke empathy were often successful at raising awareness of the struggles faced by larger people, yet largely ineffective at changing anti-fat attitudes (Daníelsdóttir et al., 2010), and resulted in worsening attitudes toward higher-weight individuals in some participants Daníelsdóttir et al., 2010; Kushner et al., 2014; Poustchi et al., 2013). It is interesting to note that such negative attitude changes may nevertheless be accompanied by increases in perceived clinical competence. A prejudice-reduction intervention study in 127 first-year medical students that involved reading a passage about weight stigma, interacting with higher-weight “standard patients”, and in-depth discussions with the standard patients, other students, and a faculty member, found that 87% of the students felt better equipped for clinical interactions with higher-weight patients, despite a third of the participants recording greater agreement with obesity stereotypes and 23% reporting less empathy toward higher-weight patients following the intervention (Kushner et al., 2014).

One possible explanation for this rebound effect may be that as weight is often considered to be largely under individual control, focussing of the hardships that higher-weight individuals face may serve to reinforce stereotypes about heavier people being weak-willed and lazy. Evidence from empirical studies suggests that perspective taking is more effective at improving attitudes in ambiguously stereotypical targets, and has little or even adverse effects when the target manifests characteristics consistent with the stereotypes of the group (Skorinko and Sinclair, 2013). This may be particularly relevant in the case of higher-weight patients presenting in a clinical setting. It is possible that this type of training may be more successful in changing attitudes toward patients whose “condition” is not usually considered self-inflicted, and this would be an interesting avenue of future research. However, in the present study, given that the majority of students were high in emotional empathy and egalitarian values when they started medical school, the fact that training was more successful in more empathic individuals is perhaps not a major cause for concern. Nevertheless, curricular activities focused on building empathy should include a component that explicitly addresses potential negative outcomes in some students.

The present study has a number of limitations. First, despite the large sample size, significant attrition occurred between baseline and follow-up, with over 20% of the original sample failing to complete the survey at the end of their fourth year. However, completers did not differ from non-completers on cognitive or emotional empathy, or on any measure of prejudice. Thus, it is unlikely that the final sample is selectively more empathic or less prejudiced. In terms of individual difference measures, students tended to be at the more “desirable” end of each scale, although the relationships between student characteristics and anti-fat attitudes were not noticeably tempered when statistically controlling for social desirability responding. Nevertheless, the skewness and low variance within the present sample may limit the applicability of these findings to the general population. However, given that the demographic characteristics of this large sample are typical of all students matriculating to U.S. medical schools in 2010 (Association of American Medical Colleges, 2008, 2011), it is likely that the findings are representative of medical students at U.S. schools.

An additional issue is that this online survey required self-report and recall on a number of measures. For example, favorability of contact with higher-weight patients, collected at year 4, may reflect more recent experiences rather than a reliable indicator of cumulative experiences. An ecological approach where impressions are recorded with every consultation or interaction may produce more reliable results, but this is unlikely to be feasible on a large scale. However, it may be possible to conduct such a study with a small sub-group on students to assess the validity of final-year impressions. Data collection at semester- or annual-intervals may be a good compromise. A similar problem may occur with regard to reported hours of training in perspective taking. Given that some curricula appear to include only a few hours of such training, accurate recall at the end of the fourth year may be unreliable. It is also possible that individual difference characteristics and prejudicial attitudes and beliefs may have resulted in variable attention to such training. Additionally, data were not collected on the methodology or content of such training, and it is likely that wide variability existed and may have had differential influence on outcomes. It may be useful to collect this information from the schools included in the study in order to compare individual differences in subjective recall, and to more thoroughly elucidate the effectiveness of training on anti-fat attitudes.

Finally, findings based upon measures of attitudes may not be generalizable to clinical encounters, although unequivocal evidence exists that negative attitudes are conveyed in both verbal and non-verbal behaviors in the healthcare setting (Zestcott et al., 2016), and the effects on doctor-patient relationships and subsequent disparities in healthcare for higher-weight patients are well-documented (Phelan et al., 2015a). Nevertheless, explicit prejudicial attitudes are only weakly associated with discriminatory behavior (Dovidio et al., 1996), and few studies have directly explored the association between physician anti-fat attitudes and clinical management in real-life settings. Additionally, individuals motivated to suppress prejudice appear able to do so given sufficient time and cognitive resources (Green et al., 2007; Plant and Devine, 2009; Burgess, 2010). Thus, raising self-awareness of implicit biases, and fostering the motivation to provide equitable care may mitigate the impact of physicians' biases on healthcare processes (van Ryn et al., 2011).

## Conclusion

The findings of the present study confirm that favorable contact with higher-weight patients is associated with more positive attitudes toward such patients after 4 years of medical school. Further, contact experiences during medical school may, in some cases, be able to overcome existing anti-fat attitudes in predicting favorability of interactions. The findings also suggest that positive contact with higher-weight patients during medical school has a stronger relationship with patient-related attitudes than with anti-fat attitudes in general. Future studies aiming to reduce prejudice in medical students should use outcome measures specific to the clinical population. Individual differences do not appear to moderate the relationship between positive patient contact and improved patient-targeted attitudes, but may alter the effectiveness of interventions designed to increase empathy. Training in perspective taking has only a weak association with attitudes toward heavier patients, and future empirical studies should focus on identifying the types of interventions that have the greatest positive impact without also driving paradoxical effects in some students.

## Ethics statement

This study was approved by the Institutional Review Boards of the Mayo Clinic and the University of Minnesota. At both timepoints, students who were invited to participant followed an email link to the consent page, which described the purpose of the study and asked them to click a button to agree to participate. If they agreed, they were sent to the survey. Students could stop the survey any time or could skip any items they wanted without financial penalty. They received $50 at each time point.

## Author contributions

SP, JD, and MvR were responsible for acquisition of the data. All authors contributed to the conception and design of the present study. AM analyzed the data. AM, SH, and SP contributed to interpretation of the data. AM drafted the initial version of the manuscript. All authors were involved in critical revision of the manuscript, approved the final manuscript, and agree to be accountable for all aspects of the work.

### Conflict of interest statement

The authors declare that the research was conducted in the absence of any commercial or financial relationships that could be construed as a potential conflict of interest.

## Footnotes

^1^Note, we use the term “higher-weight” throughout to describe heavier individuals, except when referring specifically to previous studies, scale names or survey items that use “obese” or “obesity,” or when describing BMI categories. For a discussion on terminology in weight stigma research and clinical practice, (see Meadows and Daníelsdóttir, 2016).

^2^See Supplementary Table 1 for details.

^3^See Supplementary Table 2.
