# Supplementary material for: Social Dominance Orientation, Dispositional Empathy, and Need for Cognitive Closure Moderate the Impact of Empathy-Skills Training, but Not Patient Contact, on Medical Students' Negative Attitudes toward Higher-Weight Patients
Source: Front Psychol. 2017 Apr 4;8:504. doi: 10.3389/fpsyg.2017.00504 (PMC5378792; doi:10.3389/fpsyg.2017.00504)
Supplement: Supplementary file 1 [file Table1.pdf]

Supplementary Material

Social dominance orientation, dispositional empathy, and need for cognitive closure moderate the impact of empathy-skills training, but not patient contact, on medical students' negative attitudes toward higher-weight patients

Angela Meadows*, Suzanne Higgs, Sara E Burke, John F Dovidio, Rebecca M Puhl, Michelle van Ryn, Sean M Phelan*

*** Correspondence:**

Corresponding Authors

Angela Meadows: axm583@bham.ac.uk

Sean M Phelan: phelan.sean@mayo.edu

Supplementary Table 1. Comparison of baseline scores and demographic characteristics for participants completing both Y1 and Y4 compared with those only completing Y1.

	Completers		Non-completers		
Anti-fat attitudes	N	Score	N	Score	*p*
AFAQ-Dislike	3703	2.29 (1.4)	956	2.26 (1.4)	.64
AFAQ-Fear	3703	4.54 (1.8)	956	4.54 (1.7)	.96
AFAQ-Blame	3702	3.96 (1.5)	956	3.98 (1.6)	.71
IAT	1875	0.41 (0.5)	495	0.43 (0.5)	.47
Individual differences					
Cognitive empathy	3662	5.29 (0.9)	946	5.22 (1.0)	.03
Emotional empathy	3661	5.58 (0.9)	946	5.52 (1.0)	.11
NFC-Seizing	3688	4.50 (1.0)	952	4.50 (0.9)	.87
**NFC-Freezing**	3688	2.97 (0.7)	951	3.06 (0.8)	**.00**
**SDO-Elitism**	3685	1.73 (1.1)	946	1.87 (1.1)	**.00**
SDO-Egalitarianism	3687	5.14 (1.3)	947	5.05 (1.4)	.10
Social Desirability	3676	4.20 (0.8)	946	4.22 (0.9)	.61
Demographics					
Age	3741	23.9 (2.7)	941	23.8 (2.5)	.06
Family Income	3444	7.10 (2.0)	966	7.22 (2.0)	.10
Parents' education	3719		963		.10
Graduate degree		62%		64%	
College degree		22%		23%	
No degree		16%		13%	
Gender	3756	50% male	976	50% male	.94
American	3699	85%	957	83%	.37
Race	3756		976		.02
White		65.1%		60.9%	
Other		34.9%		39.1%	
BMI	3728	23.3 (3.7)	966	23.2 (3.5)	.57
Contact with obese people before medical school					
Frequency	3677	2.87 (0.8)	947	2.83 (0.8)	.17
Favorability	3660	3.14 (0.6)	936	3.17 (0.6)	.29

AFAQ, Anti-Fat Attitudes Questionnaire; IAT, Weight Implicit Association Test; SDO, Social Dominance Orientation. Bold font indicates significant differences at $p < .05$ level.
