# Supplementary material for: Social Dominance Orientation, Dispositional Empathy, and Need for Cognitive Closure Moderate the Impact of Empathy-Skills Training, but Not Patient Contact, on Medical Students' Negative Attitudes toward Higher-Weight Patients
Source: Front Psychol. 2017 Apr 4;8:504. doi: 10.3389/fpsyg.2017.00504 (PMC5378792; doi:10.3389/fpsyg.2017.00504)
Supplement: Supplementary file 2 [file Table2.pdf]

Supplementary Material

Social dominance orientation, dispositional empathy, and need for cognitive closure moderate the impact of empathy-skills training, but not patient contact, on medical students' negative attitudes toward higher-weight patients

Angela Meadows*, Suzanne Higgs, Sara E Burke, John F Dovidio, Rebecca M Puhl, Michelle van Ryn, Sean M Phelan*

*** Correspondence:**

Corresponding Authors

Angela Meadows: axm583@bham.ac.uk

Sean M Phelan: phelan.sean@mayo.edu

Supplementary Table 2. Bivariate correlations between variables

	1	2	3	4	5	6	7	8	9	10	11	12	13	14	15	16	17	18	19	20	21	22	23
1. BMI	1	-.07§	.18§	.05‡	-.03	.15§	.07§	.09§	-.01	.05‡	.00	.03	-.01	.03	-.06‡	-.03	-.06‡	-.04‡	.02	-.06‡	-.01	-.05‡	-.06§
Y1 Anti-fat attitudes																							
2. AFAQ-Dislike		1	.20§	.42§	.11§	-.17§	-.04†	-.03	.00	-.44§	-.22§	-.23§	-.25§	.31§	-.24§	-.25§	-.34§	.05‡	.20§	-.25§	-.00	.56§	.45§
3. AFAQ-Fear of fat			1	.19§	.02	-.02	-.00	-.01	.03†	-.11§	-.04‡	-.05‡	-.08§	.07§	-.04†	-.03§	-.01	.12§	.09§	-.11§	.03	.15§	.14§
4. AFAQ-Willpower				1	.10§	-.03	.02	.02	.05‡	-.25§	-.11§	-.11§	-.17§	.26§	-.29§	-.18§	-.24§	.05‡	.17§	-.23§	-.04†	.32§	.35§
5. IAT-Weight					1	-.05†	.01	-.00	.03	-.07‡	-.05†	-.06†	-.09§	.05†	-.07‡	-.05†	-.03	.03	.07‡	-.05†	-.03	.10§	.09§
Frequency of contact with higher-weight individuals																							
6. Pre-medical school						1	.24§	.23§	.14§	.28§	.10§	.11§	.12§	-.05‡	.02	.12§	.09§	-.08§	-.12§	.03	.03	-.15§	-.15§
7. Staff, faculty, interns							1	.68§	.20§	.24§	.12§	.12§	.13§	.00	-.07§	.03	-.01	-.05‡	-.04‡	-.01	.08§	-.08§	-.09§
8. Medical students								1	.10§	.09§	.09§	.10§	.13§	.02	-.07§	.02	-.00	-.05‡	-.04†	-.01	.06§	-.05‡	-.08§
9. Patients									1	.03	.09§	.08§	.01	-.04†	.00	.06§	.03	-.05‡	-.05‡	-.02	.01	-.03	-.03
Favorability of contact with higher-weight individuals																							
10. Pre-medical school										1	.25§	.27§	.30§	-.14§	.16§	.17§	.25§	-.07§	-.17§	.19§	.02	-.33§	-.31§
11. Staff, faculty, interns											1	.82§	.65§	-.12§	.10§	.11§	.15§	-.03	-.12§	.14§	.06‡	-.29§	-.29§
12. Medical students												1	.65§	-.12§	.08†	.13§	.17§	-.03†	-.14§	.14§	.05‡	-.31§	-.30§
13. Patients													1	-.12§	.11§	.13§	.17§	-.06‡	-.13§	.16§	.07§	-.38§	-.45§
Y1 Individual difference variables																							
14. Elitism														1	-.44§	-.24§	-.36§	.09§	.25§	-.16§	.03	.23§	.21§
15. Egalitarianism															1	.29§	.37§	-.02	-.22§	.17§	-.02	-.20§	-.22§
16. Cognitive empathy																1	.47§	-.08§	-.58§	.40§	.09§	-.19§	-.20§
17. Emotional empathy																	1	.00	-.33§	.33§	.05‡	-.27§	-.28§
18. NFC – Seizing																		1	.25§	-.04†	-.00	.05‡	.06§
19. NFC - Freezing																			1	-.34§	-.05‡	.15§	.18§
20. Social desirability responding																				1	.01	-.21§	-.23§
Perspective-taking skills training																							
21. Hours of training																					1	-.02	-.45§
Y4 Anti-fat attitudes																							
22. AFAQ-Dislike																						1	.69§
23. Negative attitudes toward higher-weight patients																							1

AFAQ, Anti-fat Attitudes Questionnaire; IAT, Implicit Association Test; NFC, Need for closure

† $p < .05$

‡ $p < .01$

§ $p < .001$

frontiers
